# Application of a novel phosphinothricin *N*-acetyltransferase (RePAT) gene in developing glufosinate-resistant rice

**DOI:** 10.1038/srep21259

**Published:** 2016-02-16

**Authors:** Ying Cui, Ziduo Liu, Yue Li, Fei Zhou, Hao Chen, Yongjun Lin

**Affiliations:** 1 National Key Laboratory of Crop Genetic Improvement and National Center of Plant Gene Research, Huazhong Agricultural University, Wuhan, China; 2 National Key Laboratory of Agricultural Microbiology, Huazhong Agricultural University, Wuhan, China

## Abstract

Currently, only few glufosinate-resistant genes are available for commercial application. Thus, developing novel glufosinate-resistant genes with commercial feasibility is extremely important and urgent for agricultural production. In this study, we transferred a newly isolated *RePAT* gene into a *japonica* rice variety Zhonghua11, resulting in a large number of independent T_0_ transgenic plants, most of which grew normally under high-concentration glufosinate treatment. Four transgenic plants with one intact *RePAT* expression cassette integrated into the intergenic region were selected. Agronomic performances of their T_2_ progenies were investigated, and the results suggested that the expression of *RePAT* had no adverse effect on the agronomic performance. Definite glufosinate resistance of the selected transgenic plants was further confirmed to be related to the expression of *RePAT* by assay on the medium and qRT-PCR. The inheritance and expression of *RePAT* in two transgenic plants were confirmed to be stable. Finally, the two-year field assay of glufosinate resistance suggested that the agronomic performance of the transgenic plant (PAT11) was not affected by high dosage of glufosinate (5000 g/ha). Collectively, our study proves the high resistance of a novel gene *RePAT* to glufosinate and provides a glufosiante-resistant rice variety with agricultural application potential.

L-phosphinothricin (L-PPT) is the residue of a natural antibiotic bialaphos, which was found in the microbes *Streptomyces viridochromogenes* and *Streptomyces hygroscopicus* in the 1970s^1^. As a glutamic acid analogue, L-PPT can compete with the natural substrate of glutamine synthetase (GS) and inhibit the nitrogen-assimilation ability of GS^2^,^3^. In plants, the inhibition of GS leads to the deficiency of glutamine, the accumulation of ammonia, the indirect inhibition of photosynthesis and the final death of plant^4^. With such phytotoxicity, the ammonium salt of L-PPT (also known as glufosinate ammonium) was sold as herbicide with the trade name Basta in the 1980s, and now it is one of the most widely used nonselective herbicide.

Rice is one of the most important grain crops in the world, but rice production is confronted with the challenges of water shortage and the decrease of labor force^5^. Recently, manual transplanting is being replaced by mechanical direct seeding, which can significantly reduce the dependence on labor force but increase weed hazard and the difficulty of weed control^6^,^7^,^8^. Herbicide management is an effective way to control weed, but it has potential to damage rice. Developing herbicide-resistant rice is a way to improve the efficiency of herbicide control of weeds in rice field. Transgenic crops with resistance against several different herbicides have been approved for commercial production, among which glyphosate- and glufosinate-resistant crops are the primary types. For rice, only glufosinate-resistant varieties have been approved for food or feed.

Currently, all the glufosinate-resistant crops are developed by expressing a phosphinothricin *N-*acetyltransferase (PAT), which can detoxify L-PPT by acetylation of the amino group. The two commercially adopted glufosinate-resistant genes are *bar* and *pat*, which were isolated from *Streptomyces hygroscopicus* and *Streptomyces viridochromogenes* in 1987 and 1988 respectively^3^,^9^. Some other bacteria were also reported to have the ability to degrade or modify L-PPT, but no genes were reported to be cloned from them^10^. With the progress of genome sequencing of numerous microbes, nucleotide sequences that code a PAT family protein have been predicted in many microbes. Unfortunately, none of them was verified or applied to the development of transgenic crops. *bar* has been extensively adopted in developing glufosinate-resistant rice^11^,^12^,^13^,^14^. But it was found that different rice varieties expressing *bar* showed different levels of glufosinate resistance^11^, which was also reported in transgenic barley with *bar*^15^. Some researchers attribute this phenomenon to the differences in genetic backgrounds of the recipient plants. The proteins coded by *bar* and *pat* are highly homologous to each other, and are proved to have similar activities^16^. Thus, transgenic crops expressing *pat* may have similar problems. Considering that the PAT proteins isolated from different bacteria may have different kinetic constants and distinct glufosinate-resistances in different cellular compartments or plant cells^17^, it is highly necessary to search for novel glufosinate-resistant genes.

In our previous study, we isolated a novel PAT coding gene (*RePAT*) from the marine bacterium *Rhodococcus sp*. strain YM12^18^. The protein RePAT shows 37% identity and different kinetic constants with the protein coded by *bar* and *pat*, and has a high catalytic activity to L-PPT *in vitro*. To verify the potential of *RePAT* in developing glufosinate-resistant transgenic crops, in this study, we optimized the native nucleotide acid sequence of *RePAT* according to codon bias in rice and transferred *RePAT* into a *japonica* rice variety Zhonghua11 by *Agrobacterium*-mediated transformation. The stable integration and expression of *RePAT* in the transgenic plants were confirmed by molecular assay, while glufosinate resistance and agronomic traits of the transgenic plants were evaluated in the field. The final results showed that although the applied glufosinate dosage was as high as 5000 g/ha (corresponding to 10 times of the recommended glufosinate dosage for agricultural application), the agronomic traits of the transgenic rice expressing *RePAT* were not affected, indicating that *RePAT* is a valuable gene in developing glufosinate-resistant crops and the transgenic rice in this study is a good candidate for commercial production.

## Results

### Transformation and PCR analysis of T_0_ plants

**Figure 1:**
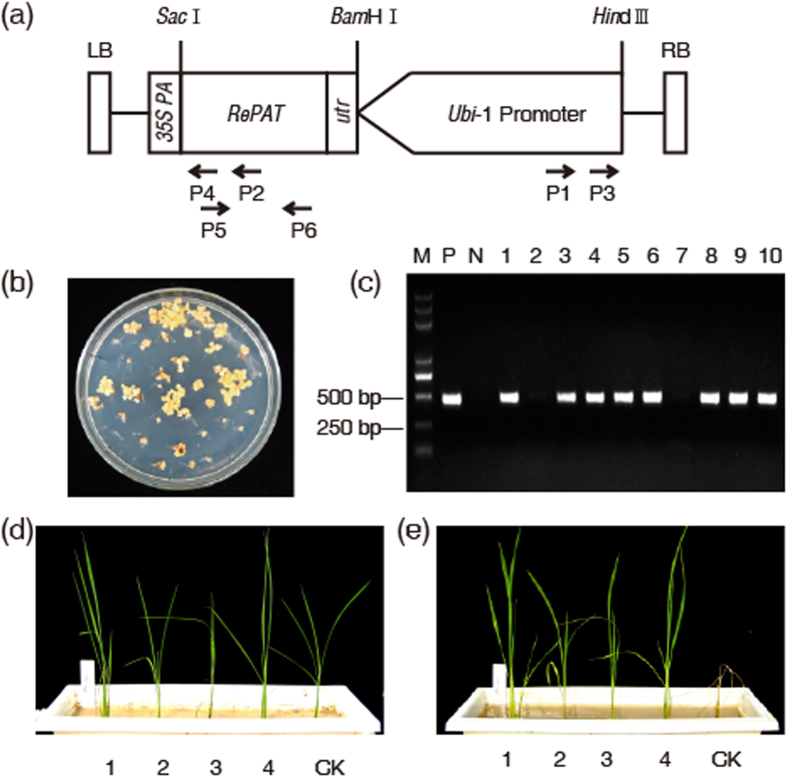
Generation and PCR detection of T_0_ transgenic rice. (**a**) T-DNA region of plant expression vector PU130 (*Ubi-*1: *RePAT*: *35S polyA*). *RePAT* was driven by maize *Ubiquitin*1 promoter and terminated by *35S PolyA*. P1, P2, P3 and P4 represent primer Ubi-1, RePAT-1, Ubi-2 and RePAT-2, respectively, which were used to separate the flanking sequence of T-DNA in rice genome with inverse PCR, while P5 and P6 are primers *RePAT*-F and *RePAT*-R for PCR assay, by which a DNA fragment with a length of 457 bp can be amplified. (**b**) Resistant calli obviously different from untransformed calli were formed in a period of 6 weeks on the medium containing 15 mg/L glufosinate. (**c**) An expected fragment with a size of 457 bp was amplified from the positive control (lane P) and eight of the ten T_0_ plants (lane 1, 3, 4, 5, 6, 8, 9 and 10) but not from the negative control (lane N) and the other two T_0_ plants (lane 2 and 7). Lane M represents 2 kb DNA marker. (**d**,**e**) were photographed 0 and 7 d after glufosinate treatment respectively. CK represents *RePAT* negative T_0_ plant, while 1–4 are *RePAT* positive T_0_ plants.

The T-DNA structure of the plant expression vector PU130 (*Ubi-*1: *RePAT*: *35S polyA*) is shown in Fig. 1a. After the calli of Zhonghua11 were infected with *Agrobacterium EHA*105 (*RePAT*), obviously resistant calli were obtained in a period of 6 weeks on the medium containing 15 mg/L glufosinate (Fig. 1b). A total of 144 independent plants were regenerated from the glufosinate-resistant calli, among which 130 independent plants had an amplified fragment with the expected size of 457 bp in the PCR assay (Fig. 1c), indicating a *RePAT* positive rate of 90% among the T_0_ plants.

### Glufosinate resistance of T_0_ plants

When glufosinate at a concentration of 1000 mg/L was sprayed over the T_0_ plants, all the *RePAT* negative plants died 7 d later, while 73% of *RePAT* positive plants grew normally without chlorosis or stunting, indicating that *RePAT* conferred glufosinate resistance to the transgenic plants (Fig. 1d,1e and Fig. S1).

### Selection of T_0_ transgenic plants with a single copy of *RePAT* integrated into the intergenic region

**Figure 2:**
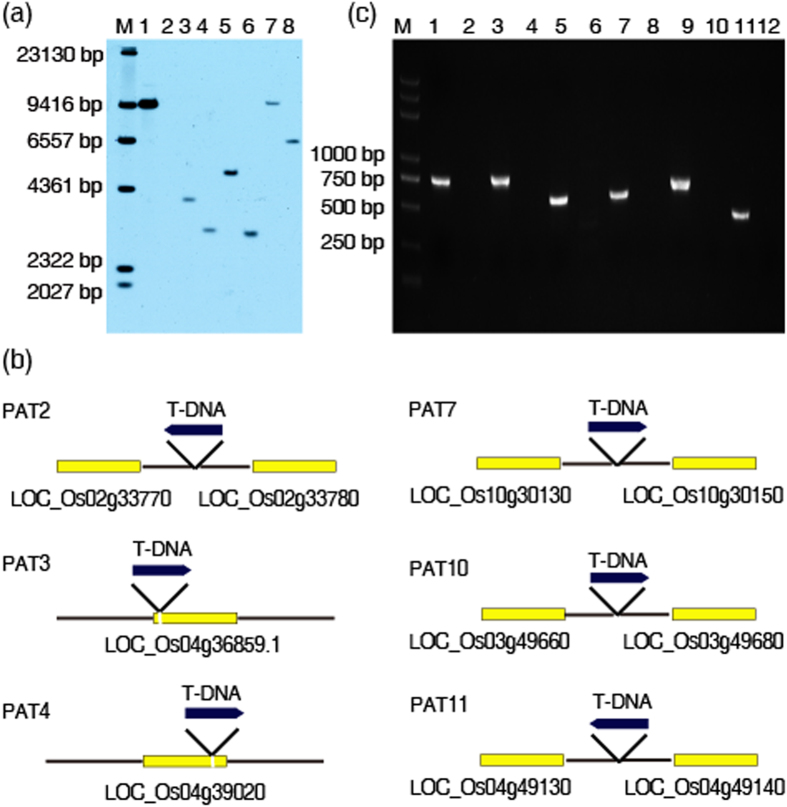
T_0_ transgenic plants with a single copy of *RePAT* integrated into the intergenic region. (**a**) In Southern blot assay, transformation vector PU130 (*Ubi-*1: *RePAT*: *35S polyA*) (lane 1) and 6 T_0_ transgenic plants (lane 3–8) had a single hybridization band, but wild type Zhonghua 11 (lane 2) had no hybridization band. Lane M is the DNA marker with the band sizes shown beside the lane. (**b**) The orientations of *RePAT* expression cassette in rice genome are indicated by the deep blue arrows which represent the direction from the right border to the left border of T-DNA. Genes nearby the integration sites and the interrupted genes are symbolized with yellow rectangles with the locus numbers of them signified below them. (**c**) The predicted integration sites were confirmed with integration-site specific PCR. Lane 1, 3, 5, 7, 9 and 11 were amplified with the DNA of PAT2, PAT3, PAT4, PAT7, PAT10 and PAT11 as templates respectively and using PAT2-R/Ubi-2, PAT3-F/Ubi-2, PAT4-R/PAT-2, PAT7-F/Ubi-2, PAT10-F/Ubi-2 and PAT11-R/Ubi-2 as primer pair for each. Lane 2, 4, 6, 8, 10 and 12 were amplified with the DNA of wild type Zhonghua 11 as template respectively and PAT2-R/Ubi-2, PAT3-F/Ubi-2, PAT4-R/PAT-2, PAT7-F/Ubi-2, PAT10-F/Ubi-2 and PAT11-R/Ubi-2 as primer pair for each. Lane M is 2 kb DNA marker. The primer sequences for integration-site specific PCR are shown in Table S1.

38 T_0_ transgenic plants with a high level of glufosinate resistance were analyzed with Southern blot, and six T_0_ transgenic plants containing a single copy of *RePAT* were selected (Fig. 2a). The six selected T_0_ transgenic plants were analyzed with inverse PCR. By running a BLAST search for the isolated flanking sequences in NCBI database, the integration site of *RePAT* expression cassette was determined (Fig. S2). The features of flanking sequences were further analyzed. The results showed that *RePAT* expression cassette in four transgenic plants (PAT2, PAT7, PAT10 and PAT11) was integrated into the intergenic region, while in the other two transgenic plants (PAT3 and PAT4) the integration sites were in the gene region (Fig. 2b). These results were further confirmed by integration-site specific PCR assay with the primer designed according to the DNA sequence nearby the predicted integration site, as specific DNA fragments with the expected sizes could be amplified from the six transgenic events but not from wild type Zhonghua11 (Fig. 2c). Finally four transgenic plants (PAT2, PAT7, PAT10 and PAT11) with *RePAT* expression cassette integrated into the intergenic region were selected for subsequent research.

### Agronomic performances of T_2_ progenies of the selected transgenic plants

**Figure 3:**
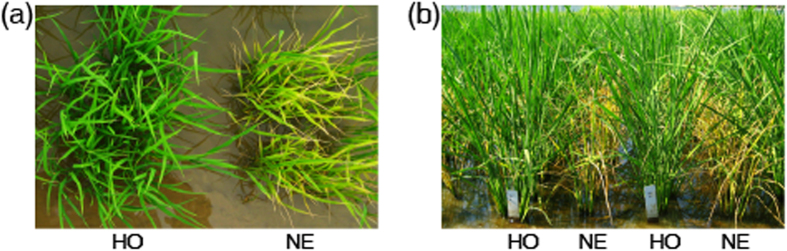
Glufosinate resistance of transgenic T_2_ progenies. (**a**) At seedling stage, 1 d after the glufosinate treatment at the concentration of 500 mg/L, negative transgenic seedlings turned yellow, while homozygous transgenic seedlings kept green. (**b**) At tillering stage, 500 mg/L glufosinate was sprayed over both the homozygous and negative transgenic plants. The homozygous transgenic rice grew without visible damage, while their corresponding negative transgenic plants completely died 7 d later. HO and NE in (**a**,**b**) represent homozygous and negative transgenic plants respectively.

Homozygous transgenic plants of PAT2, PAT7, PAT10 and PAT11 were selected according to the segregation of glufosinate resistance among T_2_ seedlings. All the seedlings of negative transgenic plants turned yellow just one day after the spraying of 500 mg/L glufosinate and completely died 7 d later; in contrast, none seedling of homozygous transgenic plants showed chlorosis symptom (Fig. 3a).

Without the glufosinate treatment, the homozygous transgenic plants of the four selected plants showed similar panicle length and filled grain rate to their corresponding negative transgenic plants. The agronomic performances of homozygous and negative transgenic plants of PAT11 showed no statistical difference to each other, but there were substantial differences in 1000-grain weight, number of panicles per plant and plant height for homozygous and negative transgenic plants of PAT2, PAT7 and PAT10 respectively (Table 1). As different homozygous transgenic plants showed variations in different aspects of agronomic traits, the expression of *RePAT* may not affect the agronomic performances, except to endow glufosinate resistance to the transgenic rice.

This inference was further confirmed by investigating the agronomic performances of homozygous and negative transgenic plants under glufosinate treatment at tillering stage. With the spraying of 500 g/ha glufosinate (at a concentration of 500 mg/L), the negative transgenic plants died completely 7 d later, while the homozygous transgenic plants grew normally without visible injury (Fig. 3b). All the treated homozygous transgenic plants were fertile and had normal agronomic performances at maturity stage (Table 2). The results of glufosinate resistance assay at seedling and tillering stage suggested that the candidate homozygous transgenic plants have obvious resistance to glufosinate.

### Expression of *RePAT* and glufosinate resistance assay on the medium

**Figure 4:**
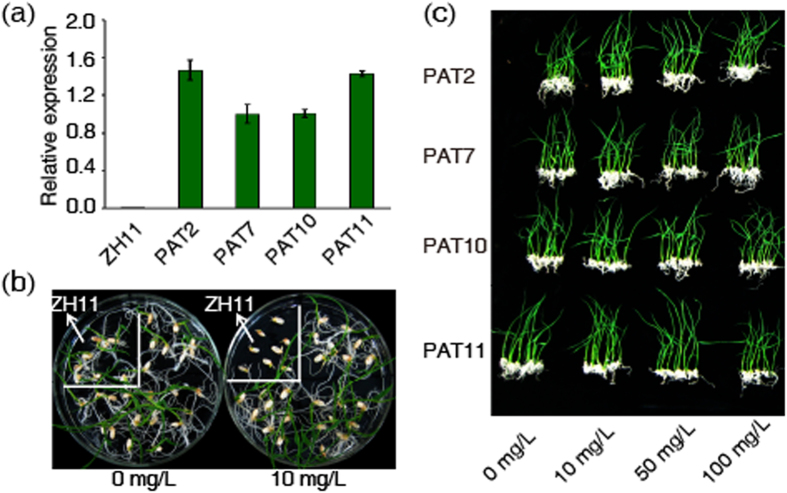
Expression of *RePAT* and glufosinate resistance assay on the medium. (**a**) Relative expression of *RePAT* in the four selected transgenic plants and wild type Zhonghua11 was determined with qRT-PCR. (**b**) On the 1/2 MS medium containing 10 mg/L glufosinate, the germination of wild type Zhonghua11 was completely inhibited, while that of transgenic plants (PAT11) was normal. (**c**) The sprouting of PAT2, PAT7, PAT10 and PAT11 on the medium containing 0, 10, 50 or 100 mg/L glufosinate was further observed, all of them were not substantially inhibited even by 100 mg/L glufosinate. (**b**,**c**) photographed 7 d and 10 d after culturing the seeds on the medium respectively. In (**a**–**c**), ZH11 represents wild type Zhonghua11, and PAT2, PAT7, PAT10, PAT11 represent the four selected transgenic plants. The concentrations of glufosinate in each test were as labeled in each figure.

Expression of *RePAT* at the transcription level was detected by qRT-PCR. Transcription of *RePAT* was not detected in wild type Zhonghua11, while was found in PAT2, PAT7, PAT10 and PAT11 (Fig. 4a). The expression of *RePAT* in the wild type and the four transgenic plants was corresponding to their glufosinate resistance on the medium. Germination of wild type Zhonghua11 was completely inhibited by 10 mg/L glufosinate on the medium (Fig. 4b), while that of transgenic rice with *RePAT* was not affected even by 100 mg/L glufosinate (Fig. 4c), suggesting that the expression of *RePAT* conferred definite glufosinate resistance to the four transgenic plants.

### Stable inheritance and expression of *RePAT* in the selected transgenic plants

**Figure 5:**
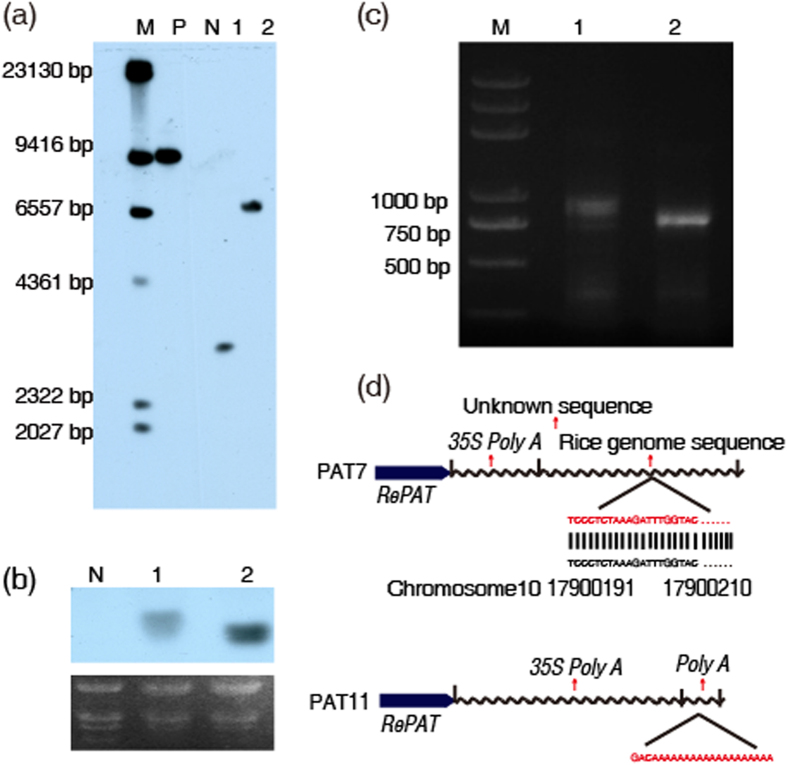
Inheritance and transcription of *RePAT* in PAT7 and PAT11. (**a**) In Southern blot assay, the hybridization band size in lane 1 (PAT7) and lane 2 (PAT11) corresponds to that in Fig. 2a (lane 6 and lane 8). Lane M is DNA marker with the band sizes shown beside the lane. (**b**) The upper panel is the hybridization result of the Northern blot. There are hybridization bands in lane 1 (PAT7) and lane 2 (PAT11) but not in lane N (wild type Zhonghua11). The size and intensity of the two hybridization bands are different. The lower panel was the RNA loading for each lane of the upper panel. (**c**) 3′ RACE products of *RePAT* transcripts in PAT7 and PAT11 are shown in this gel picture. Lane M, lane 1 and lane 2 represent 2 kb DNA marker, PAT7 and PAT11 respectively. (**d**) Compositions of the 3′ end of *RePAT* transcripts in PAT7 and PAT11 were analyzed by sequencing and a BLAST search in NCBI database. The 3′ end of *RePAT* transcript in PAT7 consists of an incomplete *35S PolyA* (with a length of 90 bp), an unknown sequence (with a length of 33 bp) and a sequence from the integration site of *RePAT* expression cassette in this plant (with a length of 214 bp). While the 3′ end of *RePAT* transcript in PAT11 is composed of an intact *35S PolyA* (with a length of 162 bp) and a poly(A) tail.

The feasibility of developing glufosinate-resistant rice with *RePAT* was further assessed with PAT7 and PAT11 as materials. At T_4_ generations, stable inheritance and expression of *RePAT* in the PAT7 and PAT11 were confirmed. In Southern blot assay, the hybridization bands of homozygous T_4_ transgenic plants of PAT7 and PAT11 were not changed compared with those of T_0_ generation (Fig. 5a), indicating that *RePAT* could be stably inherited. The transcription of *RePAT* in PAT7 and PAT11 was confirmed by Northern blot with wild type Zhonghua11 as negative control. The hybridization bands indicated that *RePAT* could be transcribed in both PAT7 and PAT11, but the transcript level and the length of transcript product were obviously different from each other (Fig. 5b). The transcription level shown by Northern blot was consistent with that displayed by qRT-PCR (Fig. 4a), indicating that the transcription level of *RePAT* in PAT7 and PAT11 was indeed different from each other. To reveal the reason for the different transcript sizes of *RePAT* in PAT7 and PAT11, *RePAT* transcripts were detected with 3′ RACE. The band size of 3′ RACE product of PAT7 was larger than that of PAT11, which is consistent with the result of Northern blot (Fig. 5c). Subsequent sequencing results showed that the 3′ end of *RePAT* transcript in PAT7 was composed of an incomplete *35S PolyA*, a short unknown sequence and a sequence from the integration site of *RePAT*, indicating that the transcription of *RePAT* was abnormally terminated in PAT7. In contrast, the 3′ end of *RePAT* transcript in PAT11 consisted of a complete *35S PolyA* and a poly(A) tail, suggesting a normal termination of *RePAT* transcription in PAT11 (Fig. 5d). Therefore, PAT11 was more suitable for developing glusfosinate-resistant rice.

### Glufosinate resistance of homozygous transgenic plants in field

In 2014, homozygous T_4_ transgenic plants of PAT11 were treated with different dosages of glufosinate both at seedling and tillering stage. At seedling stage, transgenic seedlings treated with 500, 1000, 2000, and 5000 g/ha glufosinate showed no visible injuries compared with those treated with 0 g/ha glufosinate (Fig. S3). With the application of high dosage of glufosinate at tillering stage, heading stage and pollen viability were also not substantially changed. At last, there was no substantial difference in the agronomic performance at the maturity stage under the treatments with different dosages of glufosinate (Table 3). These results indicated that PAT11 was highly resistant to glufosinate in the field. In 2015, homozygous T_6_ transgenic plants of PAT11 treated with glufosinate in the same way as in 2014 also showed no change in agronomic performances (Table 3). The two-year field assay of glufosinate resistance suggests that PAT11 is highly resistant to glufosinate and therefore has the potential to be used in agricultural production.

## Discussion

Usually, a dosage of 500 g/ha glufosinate could completely kill a non-transgenic rice. In our previous research, we determined that a wild type Zhonghua11 was sensitive to 62.5 g/ha glufosinate and was completely killed by 375 g/ha glufosinate in the field. To obtain transgenic rice highly resistant to glufosinate, we treated T_0_ transgenic plants with a high concentration of glufosinate. As more than 70% of the independent T_0_ transgenic plants with *RePAT* grew normally without chlorosis (Fig. S1), it is reasonable to infer that the high glufosinate resistance exhibited by these transgenic plants was conferred by *RePAT*. This inference was further validated by the fact that the glufosinate resistance of transgenic rice was related to the expression level of *RePAT* (Fig. S4). With significantly lower expression of *RePAT*, transgenic rice would die or show severe chlorosis under glufosinate treatment. Therefore, we speculate that the sensitivity of some transgenic rice to glufosinae is caused by low or no expression of *RePAT*.

In plant species, there are two major isoforms of GS, which are designated as GS1 and GS2 respectively. The inhibitory activities of L-PPT to GS1 and GS2 depend on the organisms from which GS1 and GS2 are isolated^19^,^20^. The location sites of GS1 and GS2 in plant cell are cytoplasm and chloroplast respectively. As the physiological environments of cytoplasm and chloroplast are different, PAT protein may have different kinetic activities in cytoplasm and chloroplast. Therefore, the isolation of PAT proteins with consistently high activity in different physiological environments will greatly enhance the feasibility of PAT in different plant species. The optimum pH of RePAT *in vitro* was proved to be 8.0^18^, which is similar to that of a methionine sulfone *N*-acetyltransferase (MAT) isolated from *Nocardia* sp^17^. Transgenic rice with such a MAT targeted to chloroplast by fusion with a chloroplast targeting signal peptide (CTP) was reported to have significantly higher glufosinate resistance than that with MAT located in cytoplasm^17^. Therefore, we propose that the addition of a CTP to RePAT may further improve the glufosinate resistance of transgenic rice. As the optimum pH for RePAT is different from that for the PAT coded by *bar* and *pat*^17^, RePAT has distinct kinetic constants. Therefore, *RePAT* is an ideal alternative to *bar* and *pat* in developing glufosinate-resistant crops.

Herbicide-resistant genes are the most important selectable marker genes in developing transgenic crops, among which *bar* has been used for more than 20 years and is still one of the most widely adopted selectable marker gene in rice transformation^21^,^22^,^23^,^24^. In our research, *RePAT* was used as the selectable marker gene in *Agrobacterium*-mediated transformation, and the results showed that a large number of glufosinate-resistant calli could be directly obtained and most of the regenerated plants from these resistant calli were *RePAT* positive, indicating that *RePAT* can be used as the selectable marker gene in place of *bar* or *pat*.

As *RePAT* is a novel glufosinate-resistant gene, to avoid the unexpected effects caused by the expression of *RePAT*, we compared the agronomic performances of homozygous T_2_ transgenic plants to those of their corresponding negative transgenic plants. Although some variations of agronomic performances were observed in homozygous transgenic plants as compared with their corresponding negative control, different homozygous plants showed variations in different aspects of agronomic performances. In particular, the variations were not related with the expression level of *RePAT*. The agronomic performances of the homozygous transgenic plants of PAT11 with relatively high expression of *RePAT* were statistically same to those of their control, suggesting that moderate expression of *RePAT* will not affect the agronomic performances of transgenic crops.

In most reports, the herbicide resistance of transgenic crops is evaluated by the occurrence of visible damage after herbicide treatment, which is far from enough to comprehensively determine the glufosinate-resistance and application potential. For example, plant height and grain yield of some glufosinate-resistant transgenic rice were reported to be affected by glufosinate^11^. Therefore, to comprehensively evaluate the glufosinate resistance of transgenic rice containing *RePAT* and select transgenic rice suitable for actual production, we not only treated the selected transgenic rice with glufosinate dosage higher than that recommended for agricultural application but also treated them both at seedling and tillering stages. We believe such treatment conditions are in line with actual production. As the major agronomic performances of the selected transgenic plant (PAT11) treated with glufosinate were comparable to those of control in two years of repeats, we suggest that the glufosinate-resistant rice that we developed here can satisfy the need of rice production. Additionally, we studied the agronomic performances of homozygous transgenic plants of PAT7 under different glufosinate treatments. Amazingly, although the transcription level of *RePAT* in PAT7 was relatively low, most agronomic performances of PAT7 were not affected by high dosages of glufosinate (Table S2), suggesting that RePAT is highly resistant to glufosinate. Collectively, we believe that the newly cloned *RePAT* is highly resistant to glufosinate and will play an important role in developing glufosinate-resistant transgenic crops.

## Methods

### Codon optimization and construction of plant expression vector

The native *RePAT* gene isolated from the marine bacterium *Rhodococcus sp*. strain YM12 has a length of 489 bp and codes 162 amino acids^18^. The sequence of *RePAT* was optimized according to codon bias in rice and fused with a 5′ untranslated region (5′ UTR) with a length of 100 bp. The fused sequence was synthesized and subsequently cloned into a modified pCAMBIA1300 vector (with deletion of the original selectable marker gene *hpt*) together with the maize *Ubiquitin1* promoter. The final plant expression vector named as PU130 (*Ubi-*1: *RePAT*: *35S polyA*) was introduced into *Agrobacterium EHA*105 by electroporation, and the recombinant *EHA*105 was designated as *EHA*105 (*RePAT*).

### *Agrobacterium*-mediated transformation

Calli induced from mature seeds of an elite *japonica* rice cultivar Zhonghua11 were used for *Agrobacterium*-mediated transformation. The procedure for inducing calli and *Agrobacterium*-mediated transformation followed the method of Hiei^25^, except that the resistant calli were selected with 15 mg/L glufosinate ammonium.

### PCR analysis of transgenic rice

Genomic DNA of transgenic rice was extracted by CTAB method^26^, and used as templates for PCR amplification. Plant expression vector PU130 (*Ubi-*1: *RePAT*: *35S polyA*) and genomic DNA of wild type Zhonghua11 were also extracted and used as templates for positive control and negative control respectively. PCR assay was performed in a mixture containing 50 ng rice genomic DNA or 1 ng plasmid DNA, 2 μL 10 × PCR buffer (Mg^2+^ plus), 0.4 μL 10 mM dNTP, 0.3 μL 10 μM *RePAT*-F (5′-GGATCCAGACTCACTCTGAG-3′), 0.3 μL 10 μM *RePAT*-R (5′-GCATGCGGTGGACACGCTGG-3′) and 1 U *Taq* DNA polymerase in a total volume of 20 μL, and under the conditions of 94 °C for 5 min, then 30 cycles of 94 °C for 30 s, 58 °C for 30 s, 72 °C for 30 s, and finally 72 °C for 8 min.

### Assay of glufosinate resistance in T_0_ transgenic plants

T_0_ transgenic plants including both PCR positive and PCR negative plants were planted into the soil. Two weeks later, they were sprayed with 1000 mg/L glufosinate solution (supplemented with 0.5% (v/v) Tween20). 7 d later, the damage of glufosinate to T_0_ transgenic plants was evaluated.

### Selection of transgenic plants with a single copy of *RePAT*

Southern blot was carried out with DIG-labeled non-radioactive detection system. 0.3 ng plant expression vector PU130 (*Ubi-*1: *RePAT*: *35S polyA*) and 10 μg genomic DNA of transgenic and wild type Zhonghua11 were digested with restriction endonuclease *Hin*d III, then separated on a 0.8% agarose gel by electrophoresis and capillary transferred onto the positively charged nylon membrane. DIG-labeled probe was prepared with PCR conditions mentioned above except that 0.01 μM DIG-dUTP was supplemented into the reaction mixture. The prehybridization, hybridization and chemiluminescent detection were performed following the DIG application manual provided by Roche Diagnostics GmbH.

### Separation of the flanking sequence of T-DNA

The integration site of *RePAT* expression cassette in the genome of transgenic rice was separated by inverse PCR. 1 μg rice genomic DNA was digested with restriction endonuclease *Hin*d III or *Sac* I, and then self-ligated with T_4_ -DNA ligase. Subsequently, two rounds of PCR were performed. In the first round of PCR, 0.5 μL of the ligation products were amplified with primer *Ubi*-1 (5′-ACTGTAGAGTCCTGTTGTCAAAATACTCAA-3′) and *RePAT*-1 (5′-ATCCACGTGATCGTCGCCTCCGTCGAGTCC-3′) in a reaction system suggested by the manufacturer of *KOD-Plus* DNA polymerase. The second round of PCR was carried out with 0.5 μL products of the first round PCR as templates and with oligonucleotide *Ubi*-2 (5′-TAGATAAACTGCACTTCAAACAAGTGTGAC-3′) and *RePAT*-2 (5′-CTACCTCCAGCTGACCCTCT-3′) as primers in a reaction system same to the first round of PCR. Products of the second round of PCR were then separated by electrophoresis, recovered, sequenced and analyzed by running a BLAST search in NCBI database and in Rice Genome Annotation Project database. The separated integration sites were further confirmed by designing primers nearby the integration sites and carrying out integration-site specific PCR.

### Selection of homozygous T_2_ transgenic plants and investigation of the agronomic performance of T_2_ progenies

The seeds of T_1_ transgenic plants with a single copy of *RePAT* integrated into the intergenic region were harvested separately and sown in the field to grow into T_2_ plants. At seedling stage, T_2_ plants were sprayed with 500 g/ha glufosinate (with a glufosiante concentration of 500 mg/L and supplemented with 0.5% (v/v) Tween 20) to identify homozygous and negative transgenic plants according to the segregation of glufosinate resistance.

10 plants of each identified homozygous or negative T_2_ transgenic plant were planted in the field with a line distance of 14 cm and a row distance of 18 cm. The field layout followed a randomized complete block design with three repetitions. At maturity 5 plants were randomly selected from each plot and the agronomic traits of them were investigated.

Homozygous and negative T_2_ transgenic plants were planted in the field in the way mentioned above. At tillering stage, these plants were treated with 500 g/ha glufosinate (with a glufosiante concentration of 500 mg/L and supplemented with 0.5% (v/v) Tween 20). 7 d later, the growth of them was observed. At maturity stage, agronomic performances of them were investigated.

### Glufosinate resistance assay on the medium

The seeds of homozygous T_3_ transgenic plants and wild type Zhonghua11 were dehulled, sterilized and transferred into the 1/2 MS medium containing different concentrations of glufosinate. They were cultured at 25 °C with 16 h light/8 h dark, and the growth of them was observed.

### Analysis of *RePAT* transcript

Total RNA of homozygous plants of the selected transgenic plants and wild type Zhonghua11 was extracted with Trizol reagent. The first strand cDNA was synthesized following the procedure provided by Invitrogen. qRT-PCR was conducted by following the manufacture’s introduction with the reagent FastStart Universal SYBR Green Master (ROX) provided by Roche. *actin* gene was used as an internal control. Primers for *RePAT* and *actin* were *RePAT*_*qrt*_-F (5′-TTCGGCTTCAGGATCGTG-3′), *RePAT*_*qrt*_-R (5′-GAGGTAGGTCATGTCGAG-3′), *actin*_*qrt*_-F (5′- AGACTACATACAACTCCATCAT-3′) and *actin*_*qrt*_-R (5′-CACCACTGAGAACGATGT-3′) respectively.

In Northern blot assay, 10 μg RNA was separated on a 1.2% formaldehyde/MOPS gel by electrophoresis and capillary transferred onto the positively charged nylon membrane. The probe for Northern blot was the same as the probe for Southern blot, and the prehybridization, hybridization and chemiluminescent detection were performed following the DIG application manual provided by Roche Diagnostics GmbH.

3′RACE was carried out following the procedure provided by Invitrogen. The first strand cDNA was synthesized as following: 3 μg RNA was mixed with 1 μL 100 μM Adapter primer (5′-GGCCACGCGTCGACTAGTACTTTTTTTTTTTTTTTTT-3′) and 1 μL 10 mM dNTP in a volume of 12 μL; then the mixture was denatured at 65 °C for 5 min and chilled on ice immediately for 3 min; subsequently 4 μL 5 × First-Strand buffer, 2 μL 0.1 M DTT, 1 μL RNaseOUT^TM^ Ribonuclease Inhibitor (40 U/μL) and 1 μL M-MLV RT were added to the mixture, which was incubated at 37 °C for 50 min; finally the mixture was heated to 70 °C for 15 min to inactivate the M-MLV RT. After that, double-strand cDNA was synthesized in a mixture of 2 μL first strand cDNA, 5 μL 10 × LA PCR buffer (Mg^2+^ plus), 1 μL 10 mM dNTP, 0.75 μL 10 μM UTR-F (5′-TTCCTTAAAGCGAAAACCCC-3′), 0.75 μL 10 μM AUAP (5′-GGCCACGCGTCGACTAGTAC-3′) and 2.5 U LA *Taq* DNA polymerase in a total volume of 50 μL, and under the conditions of 94  °C for 5 min, then 30 cycles of 94 °C for 30 s, 58 °C for 30 s, 72 °C for 60 s, and finally 72 °C for 8 min. The final PCR products were separated by electrophoresis, recovered and sequenced.

### Glufosinate resistance assay in the field

In 2014, homozygous T_4_ transgenic plants of the selected transgenic plant were sown in the field. 10 d after sowing the seedlings were sprayed with 5 different dosages of glufosinate. The 5 dosages of glufosinate were 0, 500, 1000, 2000 and 5000 g/ha respectively (the corresponding applied concentration of glufosinate to each dosage was 0, 500, 1000, 2000 and 5000 mg/L respectively, and each dosage was supplemented with 0.5% (v/v) Tween 20). 14 d later, 20 transgenic plants were randomly selected from each treatment and transplanted into the field with a line distance of 14 cm and a row distance of 18 cm, and 15 d later they were treated again with the same glufosinate dosage used at the first time. The field layout for different glufosinate treatments followed a randomized complete block design, and the treatment with each dosage was repeated 3 times. Pollen viability was evaluated by staining the pollen grains with Lugol Solution. Heading stage and other agronomic performances were investigated by following standard protocol. In 2015, homozygous T_6_ transgenic plants were planted and treated with glufosinate in the same way as in 2014, except that the plants were grown in a line distance and a row distance of 18 cm and 20 cm respectively. Agronomic performances were investigated following the method in 2014.

## Additional Information

**How to cite this article**: Cui, Y. *et al.* Application of a novel phosphinothricin N-acetyltransferase (RePAT) gene in developing glufosinate-resistant rice. *Sci. Rep.* **6**, 21259; doi: 10.1038/srep21259 (2016).

## Supplementary Material

Supplementary Information

## Figures and Tables

**Table 1:** Agronomic performances of homozygous and negative T_2_ transgenic plants.

Lines	Plant height (cm)	Panicles per plant	Panicle length (cm)	Filled grain rate (%)	1000-grain weight (g)	Yield per plant (g)
PAT2 (HO)	95.4 ± 3.2	12.0 ± 2.9	22.4 ± 0.5	58.38 ± 6.70	20.95 ± 0.31*	16.64 ± 5.43
PAT2 (NE)	88.4 ± 4.8	12.8 ± 0.7	22.1 ± 0.9	68.43 ± 6.12	19.57 ± 0.44	19.12 ± 2.12
PAT7 (HO)	99.9 ± 1.0	11.3 ± 0.8*	23.9 ± 0.5	64.75 ± 4.47	21.70 ± 0.33	18.01 ± 2.19*
PAT7 (NE)	98.1 ± 1.7	13.6 ± 0.6	23.5 ± 0.3	68.68 ± 4.94	21.73 ± 0.53	24.02 ± 1.80
PAT10 (HO)	93.1 ± 1.9*	9.6 ± 1.1	22.0 ± 1.7	68.82 ± 2.70	21.44 ± 0.74	17.28 ± 0.69
PAT10 (NE)	96.9 ± 1.3	10.3 ± 2.5	22.7 ± 0.2	71.35 ± 2.91	20.51 ± 0.16	18.18 ± 5.11
PAT11 (HO)	99.7 ± 2.9	9.4 ± 0.8	23.7 ± 0.8	74.78 ± 7.34	22.02 ± 0.51	18.36 ± 2.66
PAT11 (NE)	97.1 ± 1.8	12.0 ± 3.4	23.6 ± 1.7	69.90 ± 6.23	22.06 ± 1.55	23.09 ± 0.92

Values are means ± SD for dada collected from 5 plants in three repetitions for each plant type. “*” indicates statistically significant differences between homozygous transgenic plants and their corresponding negative transgenic plants according to *t* test (P < 0.05). “HO” and “NE” in the bracket represent homozygous and negative transgenic plants respectively.

**Table 2:** Agronomic performances of homozygous T_2_ transgenic plants under glufosinate treatment.

Homozygous lines	Plant height (cm)	Panicles per plant	Panicle length (cm)	Filled grain rate (%)	1000-grain weight (g)	Yield per plant (g)
PAT2	90.9 ± 0.6	18.4 ± 2.7	23.0 ± 0.7	50.53 ± 9.92	22.93 ± 0.60	26.64 ± 7.91
PAT7	100.4 ± 4.4	17.5 ± 2.0	23.3 ± 0.6	68.77 ± 3.36	23.06 ± 0.61	36.15 ± 3.87
PAT10	94.8 ± 0.8	14.6 ± 3.7	23.3 ± 0.6	65.00±6.58	22.10 ± 1.65	27.11 ± 2.26
PAT11	98.2 ± 5.9	15.1 ± 3.5	23.8 ± 0.9	73.92 ± 7.44	23.46 ± 0.11	32.98 ± 3.03

Values are means ± SD for dada collected from 5 plants in three repetitions for each plant type.

**Table 3:** Agronomic performances of homozygous transgenic plants of PAT11 under different glufosinate treatments in 2014 and 2015.

Year	Glufosinate dose (g/ha)	Heading date (d)	Pollen viability (%)	Plant height (cm)	Panicle length (cm)	Panicles per plant	Filled grains per plant	Filled grain rate (%)	1000-grain weight (g)	Yield per plant (g)
2014	0	71.0 ± 1.7^a^	91.59 ± 0.05^a^	111.8 ± 2.8^a^	24.4 ± 0.5^a^	8.9 ± 0.6^a^	99.8 ± 7.4^a^	78.56 ± 5.36^a^	23.96 ± 0.27^a^	21.02 ± 1.34^a^
	500	71.3 ± 2.1^a^	85.64 ± 4.86^a^	113.3 ± 1.3^a^	23.9 ± 1.1^a^	8.6 ± 0.7^a^	113.1 ± 11.6^a^	77.95 ± 5.71^a^	23.48 ± 1.33^a^	22.78 ± 4.29^a^
	1000	70.7 ± 1.2^a^	82.94 ± 2.19^a^	112.0 ± 4.4^a^	24.6 ± 0.6^a^	9.2 ± 0.3^a^	111.7 ± 3.8^a^	80.02 ± 2.20^a^	23.49 ± 0.49^a^	23.85 ± 0.83^a^
	2000	70.7 ± 1.2^a^	90.25 ± 3.50^a^	109.6 ± 1.7^a^	24.5 ± 0.2^a^	9.1 ± 0.6^a^	114.9 ± 7.5^a^	76.98 ± 2.37^a^	23.57 ± 0.31^a^	24.43 ± 1.54^a^
	5000	72.3 ± 0.6^a^	89.55 ± 3.05^a^	111.4 ± 2.3^a^	24.2 ± 0.3^a^	9.1 ± 0.7^a^	113.1 ± 4.1^a^	80.17 ± 4.79^a^	23.72 ± 0.66^a^	23.35 ± 2.50^a^
2015	0	73.0 ± 1.0^a^	94.93 ± 1.15^a^	101.9 ± 0.4^a^	23.2 ± 0.2^a^	10.4 ± 0.7^a^	95.3 ± 11.9^a^	79.63 ± 7.21^a^	25.27 ± 0.78^a^	25.06 ± 5.37^a^
	500	73.0 ± 0.0^a^	95.00 ± 2.08^a^	100.3 ± 2.2^a^	23.1 ± 0.7^a^	10.6 ± 0.7^a^	90.4 ± 11.1^a^	75.74 ± 3.23^a^	25.03 ± 0.48^a^	23.62 ± 4.57^a^
	1000	73.0 ± 0.0^a^	94.49 ± 1.11^a^	99.0 ± 1.6^a^	22.7 ± 0.5^a^	10.3 ± 0.4^a^	99.7 ± 3.2^a^	81.67 ± 3.04^a^	24.55 ± 0.28^a^	24.80 ± 1.84^a^
	2000	73.3 ± 0.6^a^	93.97 ± 2.25^a^	101.1 ± 2.6^a^	22.9 ± 0.2^a^	10.3 ± 1.5^a^	94.1 ± 19.3^a^	75.37 ± 6.55^a^	24.95 ± 1.22^a^	23.30 ± 1.12^a^
	5000	74.3 ± 1.2^a^	95.21 ± 1.89^a^	99.7 ± 2.3^a^	22.7 ± 0.2^a^	10.1 ± 0.7^a^	94.3 ± 3.8^a^	76.41 ± 6.74^a^	24.49 ± 0.47^a^	23.21 ± 3.01^a^

Values are means ± SD for dada collected from 10 plants in three repetitions for each treatment. Data followed by the same subscripted letter within a column means they are not significantly different using ANOVA analysis (P<0.05). The year of field assay was indicated in the first column.
