# Corrigendum: Application of a novel phosphinothricin *N*-acetyltransferase (RePAT) gene in developing glufosinate-resistant rice

**DOI:** 10.1038/srep46937

**Published:** 2018-01-29

**Authors:** Ying Cui, Ziduo Liu, Yue Li, Fei Zhou, Hao Chen, Yongjun Lin

Scientific Reports 6: Article number: 21259; 10.1038/srep21259 published online: 02 16 2016; updated: 01 29 2018.

This Article contains typographical errors. In Table 1, the ‘Yield per plant (g)’ for PAT11 (NE) “23.09 ± 0.92” should read “23.09 ± 8.11”. In the Methods section under subheading ‘PCR analysis of transgenic rice’,

“PCR assay was performed in a mixture containing 50 ng rice genomic DNA or 1 ng plasmid DNA, 2 μL 10 × PCR buffer (Mg^2+^ plus), 0.4 μL 10 mM dNTP, 0.3 μL 10 μM *RePAT*-F (5′-GGATCCAGACTCACTCTGAG-3′), 0.3 μL 10 μM *RePAT*-R (5′-GCATGCGGTGGACACGCTGG-3′) and 1 U *Taq* DNA polymerase in a total volume of 20 μL, and under the conditions of 94 °C for 5 min, then 30 cycles of 94 °C for 30 s, 58 °C for 30 s, 72 °C for 30 s, and finally 72 °C for 8 min.”

should read:

“PCR assay was performed in a mixture containing 50 ng rice genomic DNA or 1 ng plasmid DNA, 2 μL 10 × PCR buffer (Mg^2+^ plus), 0.4 μL 10 mM dNTP, 0.3 μL 10 μM *RePAT*-F (5′-TTCCTTAAAGCGAAAACCCC-3′), 0.3 μL 10 μM *RePAT*-R (5′-ACGATCCTGAAGCCGAACCT-3′) and 1 U *Taq* DNA polymerase in a total volume of 20 μL, and under the conditions of 94 °C for 5 min, then 30 cycles of 94 °C for 30 s, 58 °C for 30 s, 72 °C for 30 s, and finally 72 °C for 8 min.”

