# Supplementary material for: Application of a novel phosphinothricin N-acetyltransferase (RePAT) gene in developing glufosinate-resistant rice
Source: Sci Rep. 2016 Feb 16;6:21259. doi: 10.1038/srep21259 (PMC4754654; doi:10.1038/srep21259)
Supplement: Supplementary Information [file srep21259-s1.pdf]

Application of a novel phosphinothricin *N*-acetyltransferase (RePAT) gene in developing glufosinate-resistant rice

Ying Cui[1], Ziduo Liu[2], Yue Li[1], Fei Zhou[1], Hao Chen[1], Yongjun Lin[1*]

[1]National Key Laboratory of Crop Genetic Improvement and National Center of Plant Gene Research, Huazhong Agricultural University, Wuhan, China

[2]National Key Laboratory of Agricultural Microbiology, Huazhong Agricultural University, Wuhan, China

[*]Corresponding author: Tel/fax 86-027-87281719; email yongjunlin@mail.hzau.edu.cn

Supplementary Figure S1.

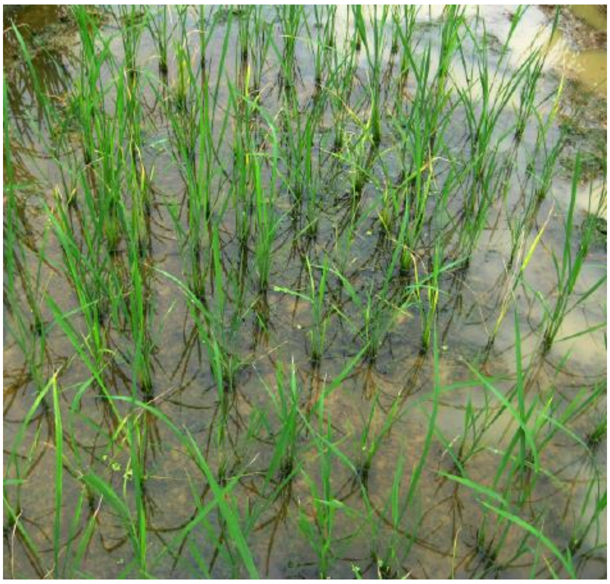

Fig. S1 Glufosinate resistance of T_0 transgenic plants in the field. T_0 transgenic plants were transferred into the field. Most T_0 transgenic plants showed normal phenotype under the treatment with 1000 mg/L glufosinate.

Supplementary Figure S2.

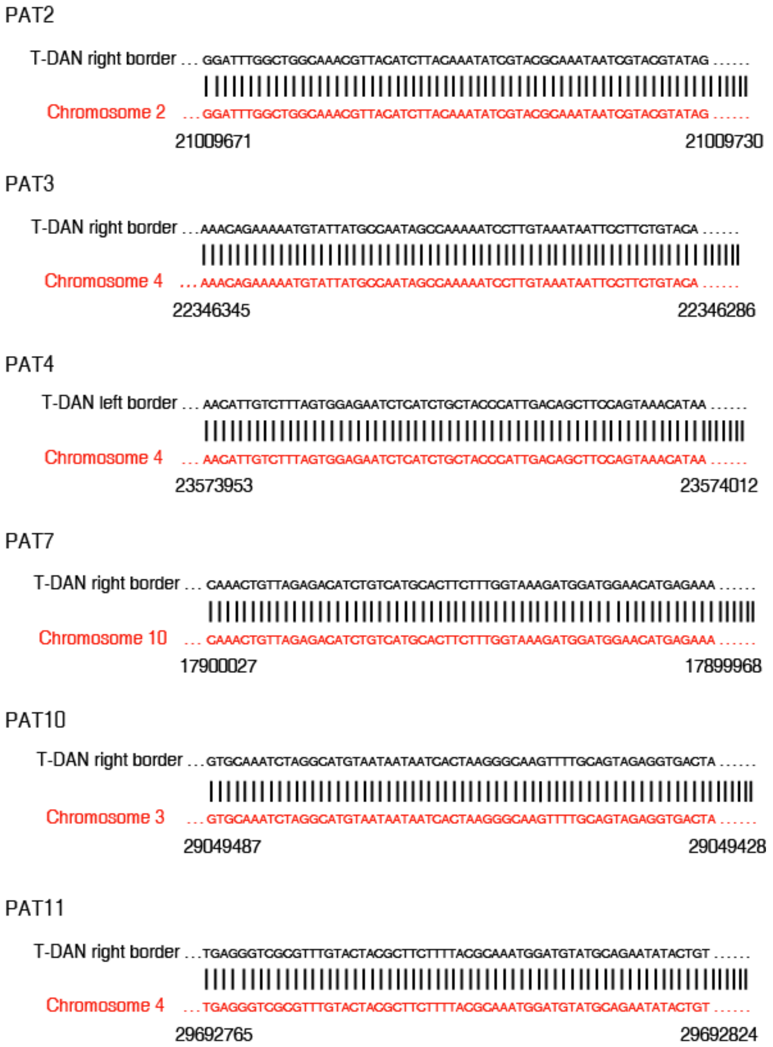

Fig. S2 Integration sites of the *RePAT* expression cassette in rice genome. The sequences of the fragments isolated by inverse PCR were analyzed by a BLAST search in NCBI database, and the integration sites are indicated by the matching position of the isolated sequences to rice genome. DNA sequences with black color and red color represent the isolated flanking sequences of *RePAT* expression cassette and rice genome sequences respectively.

Supplementary Figure S3.

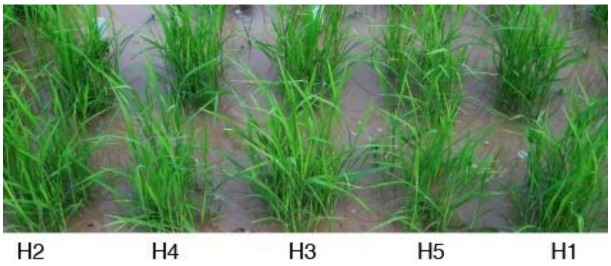

Fig. S3 Glufosinate resistance of homozygous T_4 transgenic seedlings. The growth of the seedlings was photographed 7 d after the treatment with different dosages of glufosinate. H1, H2, H3, H4 and H5 represent the dosage 0, 500, 1000, 2000 and 5000 g/ha respectively.

Supplementary Figure S4.

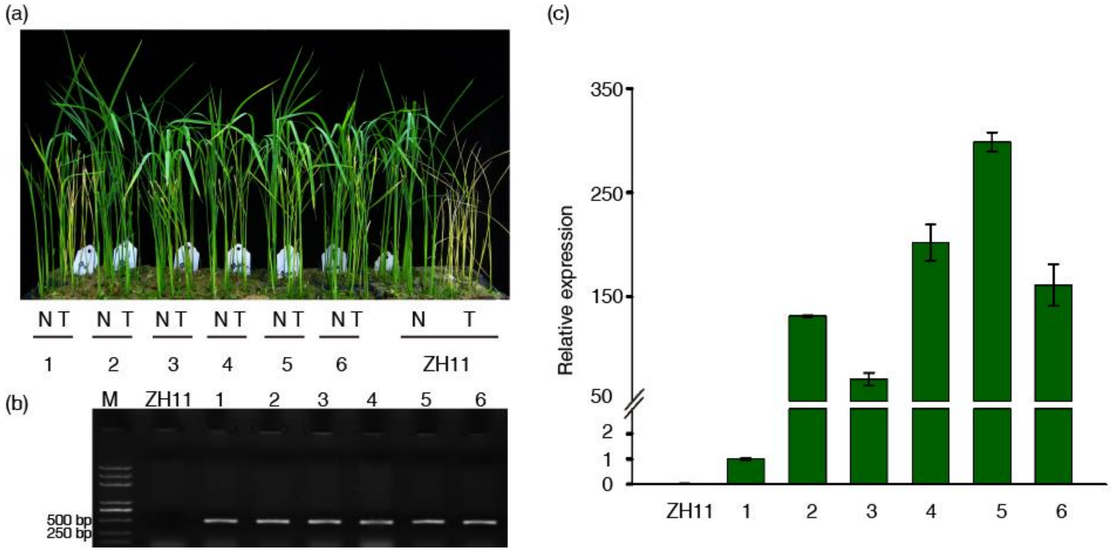

Fig. S4 Expression of *RePAT* and glufosinate resistance assay. (a) At seedling stage, transgenic plants (1 to 6) and wild type Zhonghua11 (ZH11) were treated with 0 or 5000 mg/L glufosinate. 7 d later, under the treatment of 5000 mg/L glufosinate, wild type Zhonghua11 completely died, while transgenic plants with different expression level of *RePAT* showed different phenotype. "N" represents transgenic plants and wild type Zhonghua11 treated with 0 mg/L glufosinate, and "T" represents transgenic plants and wild type Zhonghua11 treated with 5000 mg/L glufosiante. (b) PCR assay of the tested plants. *RePAT* was amplified from transgenic plants (1 to 6) but not from Zhonghua 11 (ZH11). (c) Q-PCR was performed to detect the expression of *RePAT* in 6 different transgenic plants (1 to 6) and wild type Zhonghua11 (ZH11). Transgenic plants 1 had significantly lower expression of *RePAT* than the other transgenic plants.

Supplementary Table S1. Primer sequences for integration-site specific PCR.

Primers	Sequences
PAT2-F	CAGCGATAAGCATGGCAACC
PAT2-R	GACCTGAGTTGCATCATCAG
PAT3-F	GCACATTCACTCCTCCTGAC
PAT3-R	GTCCATGTGACTATGAGACG
PAT4-F	GCATACCATGTCCCCAGCTG
PAT4-R	GCTCTCCTATGCTGCTAACC
PAT7-F	CTTCTACACCAACTGGGGTG
PAT7-R	CTCACGAAGGACAATCTAGC
PAT10-F	GCATGCTGTGACGTGAACTC
PAT10-R	TGACATCACCCATGGAGATC
PAT11-F	CACCTAGGATGATGACGTGG
PAT11-R	CGCTAACAGTGCTAACATTC

Supplementary Table S2. Agronomic performances of homozygous transgenic plants of PAT7 with different glufosinate treatments in 2014.

Homozygous plants	Glufosinate dose (g/ha)	Heading date (d)	Pollen viability (%)	Plant height (cm)	Panicle length (cm)	Panicles per plant	Filled grains per plant	Filled grain rate (%)	1000-grain weight (g)	Yield per plant (g)
	0	69.3±0.6[a]	90.66±2.57[a]	110.1±3.7[a]	24.3±0.1[a]	9.3±1.1[a]	110.0±7.8[a]	80.78±0.50[a]	24.38± 0.57[a]	24.64±3.81[a]
	500	70.7±2.5[a]	90.39±0.84[a]	110.9±0.8[a]	24.1±0.3[a]	9.5±0.9[a]	108.1±4.4[a]	77.00±4.32[a]	23.93± 0.26[a]	24.12±1.15[a]
PAT7	1000	71.3±1.5[a]	84.25±8.07[a]	107.5±5.2[a]	23.9±0.3[ab]	8.0±1.1[a]	102.6±4.3[a]	80.49±4.88[a]	24.26±0.92[a]	20.09±2.91[a]
	2000	70.7±0.6[a]	87.32±2.39[a]	108.1±2.3[a]	23.3±0.1[bc]	8.5±1.1[a]	107.2±5.7[a]	80.38±2.18[a]	24.03±0.68[a]	21.75±2.10[a]
	5000	73.0±3.0[a]	79.03±7.94[a]	111.2±3.6[a]	23.7±0.1[c]	9.1±0.4[a]	102.2±3.4[a]	82.10±3.73[a]	24.22±0.20[a]	22.34±0.72[a]

Values are means±SD for dada collected from 10 plants in three replications for each treatment. Different subscripted letters (a-c) within the column indicate statistically significant differences among the treatments according to least significant difference method ($P<0.05$).
